# Analysis of the physical properties of spindle seeds for seed sorting operations

**DOI:** 10.1038/s41598-021-93166-z

**Published:** 2021-07-01

**Authors:** Zdzisław Kaliniewicz, Andrzej Anders, Piotr Markowski, Paweł Tylek, Danuta Owoc

**Affiliations:** 1 Department of Heavy Duty Machines and Research Methodology, University of Warmia and Mazury in Olsztyn, ul. Oczapowskiego 11, 10-719 Olsztyn, Poland; 2 Faculty of Forestry, University of Agriculture in Cracow, Al. 29 Listopada 46, 31-425 Kraków, Poland

**Keywords:** Biotechnology, Plant sciences, Engineering

## Abstract

The relationships between the basic physical properties of seeds of selected spindle species were evaluated for the needs of seed sorting operations. Physical properties were measured in the seeds of five spindle species, and the presence of relationships between these attributes was determined in correlation and regression analyses. The average values of the evaluated parameters were determined in the following range: terminal velocity—from 9.2 to 10.3 m s^−1^, thickness—from 2.57 to 3.26 mm, width—from 2.87 to 3.74 mm, length—from 3.94 to 5.52 mm, angle of external friction—from 20.7° to 24.6°, mass—from 16.5 to 33.8 mg. Spindle seeds were arranged in the following ascending order based on their geometric mean diameter: winged spindle, Hamilton’s spindle, large-winged spindle, broadleaf spindle and European spindle. Spindle seeds should be separated in a sieve equipped with at least two mesh screens with slotted apertures. Depending on the processed spindle species, aperture size should range from ≠ 2.7 to ≠ 3.5 mm in the top screen, and from ≠ 2.4 to ≠ 3.0 mm in the bottom screen.

## Introduction

Shrubs play very important roles in forest ecosystems by improving the quality of the local environment, enhancing species composition and stabilizing soils^1–5^. Their beneficial effects are particularly visible in nutrient-deficient habitats, where shrubs and tree stands can enrich the composition of plant litter, promote its humification and mineralization, and contribute to soil fertility. Shrubs protect tree stands against winds and act as a food source for forest fauna^2,5–7^. Shrubs are ornamental plants with multi-colored stems, flowers, leaves and fruit, which enhance the scenic value of forests and forest boundary zones^1,2,8^. Shrubs are also expansive plants, and according to estimates, they can occupy up to 45% of the Earth’s total land surface area^7,9^. Shrubs are increasingly often used in forest management, and they attract considerable interest on account of their biological and environmental properties. They are also a valuable source of raw materials for pharmaceutical and food processing industries^10–12^.

Spindles are shrubs, small trees, ground cover plants or woody climbers that belong to the genus *Euonymus*. They are found in North and Central America, Europe, Asia, Central Africa and Australia, where they grow mainly on fertile, fresh and humus-rich soils^10,13–16^. Around 130 spindle species have been identified to date, and many of them contain strongly toxic substances^10,13^. Most spindles tolerate cutting and pruning, and they are suitable for hedging. Spindles produce colorful capsular fruit with up to five carpels containing 1–4 seeds each. The seeds of most spindle species are poisonous for humans^10,13,15^. Spindle fruit are consumed by birds which digest only arils and excrete seeds with feces, thus contributing to the spread of this group of plants^10,14^. The seeds and stems of some spindle species are a source of biologically active substances such as diterpenes, triterpenes, sesquiterpenes, cardiac glycosides, lectins, alkaloids and squalene^17–21^. Tinctures made of spindle seeds have antioxidant, antibacterial and insecticidal properties, and they are used for detoxification and in the treatment of cancer, hyperglycemia, menstrual discomfort, diabetic complications, and dermatological diseases^16,17,21–23^.

Spindle seeds differ considerably due to the multitude of *Euonymus* species that occupy various regions of the world and adapt to local conditions. Therefore, there is no single method for preparing the seeds of this genus for sowing, and seeds of different species have to be processed independently. During processing, seeds should be sorted into groups characterized by high and similar germination capacity. According to research^24–29^, germination rates are directly correlated with seed mass, but it is not always linked with seed size. In rare cases, small seeds germinate faster than larger seeds^30–32^. Seeds are sorted into fractions with specific seed mass, and fractions are sown separately to ensure even seedling emergence, to facilitate agronomic treatments and produce plants which a uniform habit that are easy to plant mechanically^33,34^. However, effective industrial methods for sorting seeds into mass fractions have not been developed to date. Seeds are separated with the use of vibrating screens, combined with a stream of air in some devices. The separated seeds should have different dimensions and similar mass or, conversely, similar dimensions and different mass to guarantee the effectiveness of the sorting process^35^. Seeds that differ in both mass and dimensions cannot be accurately sorted into fractions. Therefore, the relationships between seed mass and other physical properties have to be analyzed to effectively plan and perform seed cleaning and sorting operations^34–37^. There is a general scarcity of published data on the distribution of basic physical properties of seeds belonging to different spindle species. Such information is essential not only for sorting, but also for harvesting, storing, sowing and processing seeds.

In view of the above, the aim of this study was to determine the relationships between the basic physical properties of the seeds of various spindle species for the purpose of planning and performing seed sorting operations.

## Results and discussion

### Experimental material

The evaluated samples comprised 107–119 seeds. Based on the standard deviation of the analyzed properties and the values of Student’s t-distribution at a significance level of α = 0.05, the standard error of the estimate did not exceed:

- 0.2 m s^−1^ for terminal velocity,
- 0.1 mm for seed thickness and width,
- 0.2 mm for seed length,
- 1° for the angle of external friction,
- 2 mg for seed mass.

The physical properties of seeds of selected spindle species are presented in Table 1. Average terminal velocity ranged from 9.2 m s^−1^ (winged spindle) to 10.3 m s^−1^ (European spindle), and similar values have been reported in pea seeds^38^, cowpea seeds^39^ and common beech seeds^40^. The seeds of broadleaf, large-winged and Hamilton’s spindle formed a homogeneous group in terms of terminal velocity.

**Table 1:** Mean values ± standard deviation of the distribution of the physical properties of spindle seeds, with an indication of significant differences.

Property/indicator	Spindle species				
	Broadleaf	Large-winged	European	Hamilton’s	Winged
Terminal velocity *v* (m s^−1^)	9.5 ± 0.6^b^	9.7 ± 0.7^b^	10.3 ± 0.8^c^	9.6 ± 0.8^b^	9.2 ± 1.0^a^
Thickness *T* (mm)	2.98 ± 0.30^c^	2.83 ± 0.34^b^	3.26 ± 0.34^d^	2.61 ± 0.26^a^	2.57 ± 0.36^a^
Width *W* (mm)	3.41 ± 0.30^d^	3.20 ± 0.37^c^	3.74 ± 0.37^e^	2.97 ± 0.26^b^	2.87 ± 0.30^a^
Length *L* (mm)	5.52 ± 0.50^c^	5.05 ± 0.54^b^	5.41 ± 0.73^c^	5.09 ± 0.62^b^	3.94 ± 0.49^a^
Angle of external friction *γ* (°)	24.6 ± 3.2^b^	23.3 ± 3.0^b^	20.7 ± 3.2^a^	21.6 ± 4.6^a^	23.6 ± 4.6^b^
Mass *m* (mg)	26.1 ± 4.7^d^	23.5 ± 5.5^c^	33.8 ± 8.4^e^	20.3 ± 5.0^b^	16.5 ± 5.0^a^
Aspect ratio *T*/*W* (%)	87.7 ± 9.0^a^	88.9 ± 8.6^a^	87.5 ± 7.8^a^	87.9 ± 7.7^a^	89.7 ± 9.0^a^
Aspect ratio *T*/*L* (%)	54.2 ± 5.3^b^	56.5 ± 7.8^c^	60.8 ± 6.2^d^	51.7 ± 5.7^a^	66.2 ± 9.8^e^
Aspect ratio *W*/*L* (%)	62.0 ± 5.3^b^	63.7 ± 7.3^b^	69.8 ± 7.6^c^	59.2 ± 7.8^a^	73.8 ± 9.6^d^
Geom. mean diameter *D* (mm)	3.82 ± 0.29^d^	3.57 ± 0.33^c^	4.03 ± 0.40^e^	3.40 ± 0.29^b^	3.06 ± 0.30^a^
Sphericity index *Φ* (%)	69.4 ± 3.5^b^	71.0 ± 5.5^c^	75.0 ± 4.7^d^	67.3 ± 4.9^a^	78.5 ± 7.4^e^
Specific mass *m*_*D*_ (g m^−1^)	6.8 ± 0.8^d^	6.5 ± 1.0^c^	8.3 ± 1.3^e^	5.9 ± 1.1^b^	5.3 ± 1.2^a^

a, b, c—superscript letters denote significant differences between the examined properties.

Winged spindle was characterized by the smallest seeds, whereas European spindle seeds were largest. The average values of basic seed dimensions were determined in the following range: thickness—2.57 to 3.26 mm, width—2.87 to 3.74 mm, length—3.94 to 5.52 mm. Seeds with similar thickness and width were noted in selected cereal species^41–45^, whereas seeds with similar width and length were reported in Scots pine^46–48^ and bishop pine^49^. Hamilton’s and winged spindle seeds formed a homogeneous group in terms of seed thickness. Large-winged and European spindle seeds were similar in width. The seeds of large-winged, Hamilton’s, broadleaf and European spindle formed a homogeneous group in terms of length.

The average angle of external friction ranged from 20.7° (European spindle) to 24.6° (broadleaf spindle), and the evaluated species were divided into two homogeneous groups based on this trait. The above values corresponded to the coefficients of external friction in the range of 0.38 to 0.46. The porosity of the friction plate is rarely given in the literature. In studies evaluating seeds with a similar moisture content on a steel friction plate (whose porosity is unknown), similar values of the coefficient of external friction were reported by Omobuwajo et al.^50^ in ackee apple seeds, Konak et al.^51^ in chick pea seeds, Ogunjimi et al.^52^ in locust bean seeds, Bart-Plange and Baryeh^53^ in cocoa beans, Amin et al.^54^ in lentil seeds, Altuntaş et al.^55^ in fenugreek seeds, and Kaliniewicz et al.^45^ in the seeds of the major cereal species.

Seed mass is influenced mainly by basic seed dimensions, and it was highest in European spindle (33.8 mm) and lowest in winged spindle (16.5 mg). The studied spindle species differed significantly in seed mass and geometric mean diameter. In terms of mass, European spindle seeds are similar to field maple and small-leaved lime seeds^56^ and the seeds of selected wheat cultivars^43,44^; broadleaf spindle seeds most closely resemble European ash seeds^56^ and grand fir seeds^37^; large-winged spindle seeds are similar to glossy buckthorn seeds^56^; Hamilton’s spindle seeds resemble common ivy seeds^56^, European black pine seeds^34^ and Japanese fir seeds^37^; and winged spindle seeds are similar to corkbark fir seeds^37^.

The physical properties of seeds are also influenced by their relative moisture content. According to many authors^38,39,51,54,55^, terminal velocity, seed dimensions, seed mass and the angle of external friction increase with a rise in the relative moisture content of seeds. However, seeds of the analyzed species are considered orthodox, and they have to be significantly dehydrated immediately after harvest and before storage. For this reason, relative moisture content was not considered as a variable in this study.

The seeds of the evaluated spindle species did not differ significantly in the *T/W* aspect ratio which ranged from 88 to 90%. Spindle seeds were more diverse in terms of the remaining aspect ratios, and the only homogeneous group was formed by broadleaf and large-winged spindle seeds which were characterized by a similar *W/L* aspect ratio. Broadleaf spindle seeds are similar to pedunculate oak seeds^57^ in terms of the *T/L* aspect ratio, and to African star apple seeds^58^ in terms of the *W/L* aspect ratio. As regards the *W/L* aspect ratio, Hamilton’s seeds resemble balsam and Korean fir seeds^37^, and European spindle seeds are similar to common hornbeam and black locust seeds^59^. Winged spindle seeds and cowpea seeds^39^ are characterized by similar values of the sphericity index.

Specific seed mass was determined in the range of 5.3 g m^−1^ (winged spindle) to 8.3 g m^−1^ (European spindle), and the analyzed species differed significantly in this trait. In terms of specific mass, broadleaf spindle seeds are highly similar to Forrest’s fir seeds^37^; European spindle seeds closely resemble silver and white fir seeds^37^; Hamilton’s spindle seeds are similar to small-leaved lime seeds^59^ and Sierra white fir seeds^37^; and winged spindle seeds resemble Morinda spruce seeds^60^ and European black pine seeds^34^.

The analysis of basic seed dimensions revealed the highest number of similarities between broadleaf and large-winged spindle seeds (which formed homogeneous groups in terms of for traits) and the fewest similarities between large-winged and European spindle seeds, and between European and winged spindle seeds (which formed a homogeneous group based on a single trait). European spindle seeds differed most considerably from the remaining spindle species (6 similarities), whereas broadleaf, large-winged and Hamilton’s spindle seeds were most similar to the remaining species (10 similarities each).

### Correlations between seed properties

The results of the correlation analysis evaluating the strength of the relationships between the physical properties of seeds that can be potentially used in sorting processes are presented in Table 2. The value of the correlation coefficient was practically significant (above 0.4) in 53 out of 90 comparisons. The highest number of correlations was noted between seed mass and the remaining seed properties (26 comparisons), whereas the angle of external friction was the least correlated with the remaining seed properties (7 comparisons). The highest (0.909) and lowest (0.004) values of the correlation coefficient were noted in European spindle seeds in comparisons of seed mass vs. seed length, and seed mass vs. the angle of external friction, respectively. The strongest correlations between seed mass and seed length were observed in broadleaf, European and Hamilton’s spindle seeds. Seed mass and seed width were bound by the strongest correlations in large-winged and winged spindle seeds. When the seeds of the evaluated spindle species were pooled into a single group, the highest values of the correlation coefficient were noted in comparisons of seed mass and seed width, followed by comparisons of seed mass and seed thickness. The lowest values of the correlation coefficient (which were statistically, but not practically significant) were obtained in comparisons of the angle of external friction with the remaining seed properties.

**Table 2:** Coefficients of linear correlation between the basic physical properties of spindle seeds.

Spindle species		Properties				
		*v*	*T*	*W*	*L*	*γ*
Broadleaf	T	**0.401**	1			
	*W*	**0.193**	**0.360**	1		
	*L*	0.073	**0.454**	**0.576**	1	
	*γ*	**− 0.223**	− 0.095	− 0.064	0.074	1
	*m*	**0.403**	**0.706**	**0.684**	**0.804**	− 0.011
Large-winged	*T*	**0.654**	1			
	*W*	**0.566**	**0.629**	1		
	*L*	**0.399**	**0.302**	**0.500**	1	
	*γ*	**− 0.476**	**− 0.428**	**− 0.383**	**− 0.305**	1
	*m*	**0.665**	**0.779**	**0.795**	**0.749**	**− 0.438**
European	*T*	**0.406**	1			
	*W*	**0.198**	**0.599**	1		
	*L*	**0.329**	**0.664**	**0.625**	1	
	*γ*	0.083	− 0.084	**0.243**	− 0.110	1
	*m*	**0.425**	**0.819**	**0.777**	**0.909**	0.004
Hamilton’s	*T*	**0.637**	1			
	*W*	**0.363**	**0.531**	1		
	*L*	**0.619**	**0.616**	**0.375**	1	
	*γ*	**− 0.564**	**− 0.360**	0.063	**− 0.432**	1
	*m*	**0.694**	**0.803**	**0.594**	**0.878**	**− 0.412**
Winged	*T*	**0.462**	1			
	*W*	**0.343**	**0.682**	1		
	*L*	0.072	**0.242**	**0.396**	1	
	*γ*	**− 0.368**	**− 0.504**	**− 0.220**	**0.215**	1
	*m*	**0.654**	**0.691**	**0.708**	**0.602**	**− 0.204**
Total	*T*	**0.584**	1			
	*W*	**0.472**	**0.751**	1		
	*L*	**0.395**	**0.567**	**0.627**	1	
	*γ*	**− 0.381**	**− 0.296**	**− 0.133**	**− 0.130**	1
	*m*	**0.697**	**0.846**	**0.853**	**0.792**	**− 0.240**

*v* terminal velocity, *T* thickness, *W* width, *L* length, *γ* angle of external friction, *m* mass.

Values in bold indicate statistically significant correlations.

**Figure 1:**
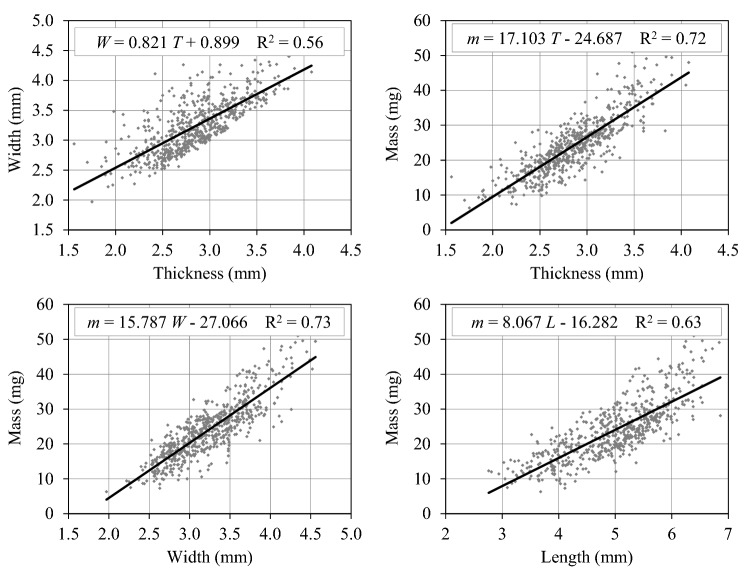
Relationships between the physical properties of seeds.

The above results were confirmed by regression analysis (Fig. 1) where the coefficient of determination for the compared properties did not exceed 0.5. The coefficient of determination exceeded 0.5 in four linear regression relationships, and it was highest (R^2^ = 0.73, F(1,581) = 1546.5) for the relationship between seed width and seed mass. Seed mass increased by more than 1000% (from approx. 4 mg to approx. 45 mg) when seed width increased from around 2.0 mm to around 4.6 mm. The coefficient of determination was also high (R^2^ = 0.72, F(1,581) = 1458.4) for the relationship between seed thickness and seed mass. These results indicate that spindle seeds should be separated with the use of mesh screens with round or slotted apertures.

### Recommendations for seed sorting

A thorough knowledge of variations in the physical properties of the analyzed materials and the correlations between these properties is required to effectively plan and model sorting and processing operations^35^. The above also applies to seeds. The seeds of the evaluated spindle species were divided into three classes based on differences in their dimensions and mass. The boundary values for separating seeds into classes differed in each spindle species (Table 3). Seeds lighter than 14 mg (winged spindle), 18 mg (Hamilton’s spindle), 20 mg (large-winged spindle), 24 mg (broadleaf spindle) and 28 mg (European spindle) were classified as the smallest seeds (class 1). In turn, seeds heavier than 18 mg (winged spindle), 22 mg (Hamilton’s spindle), 26 mg (large-winged spindle), 28 mg (broadleaf spindle) and 38 mg (European spindle) were classified as the largest seeds (class 3). The percentage distribution of seeds in each fraction ranged from 27.6 to 37.9% (class 1 and 2 Hamilton’s spindle seeds, respectively).

**Table 3:** Seed mass classes in the examined spindle species.

Spindle species	Seed class	Percentage (%)
Broadleaf	1 (*m* < 24 mg)	30.2
	2 (*m* = 24–28 mg)	37.0
	3 (*m* > 28 mg)	32.8
Large-winged	1 (*m* < 20 mg)	30.3
	2 (*m* = 20–26 mg)	35.3
	3 (*m* > 26 mg)	34.4
European	1 (*m* < 28 mg)	30.7
	2 (*m* = 28–38 mg)	34.2
	3 (*m* > 38 mg)	35.1
Hamilton’s	1 (*m* < 18 mg)	27.6
	2 (*m* = 18–22 mg)	37.9
	3 (*m* > 22 mg)	34.5
Winged	1 (*m* < 14 mg)	33.1
	2 (*m* = 14–18 mg)	33.1
	3 (*m* > 18 mg)	33.8

*m*—mass.

As previously noted, the smallest value of the correlation coefficient was noted in the relationship between the angle of external friction and the remaining seed properties. This result indicates that seeds of forest trees and shrubs should not be separated based on their frictional properties and that this trait should be used only as an auxiliary feature during seed separation. Similar observations made by Tylek^61^ and by Kaliniewicz and Tylek^34^ who examined the physical properties of common beach and black pine seeds. The strongest correlations were noted between two basic dimensions (width and thickness) and seed mass, which indicates that spindle seeds should be separated with the use of mesh screens with round and slotted apertures. However, broadleaf, European and Hamilton’s spindle seeds can also be sorted in cylindrical separators. In most cases (excluding the thinnest fraction of broadleaf spindle seeds), the separation of seeds into three fractions based on seed width and thickness (Table 4) decreased the coefficient of variation of seed mass in a given fraction. The obtained fractions were more uniform in terms of seed mass, and seed mass was generally most uniform in fractions with the thickest or widest seeds. In these fractions, the coefficient of variation of seed mass was lower than the coefficient of variation of seed mass in the entire seed lot, in the range of around 23% (widest winged spindle seeds) to 45% (thickest Hamilton’s spindle seeds). A comparison of variations in seed mass in different size fractions revealed that the optimal ratio of coefficients of variation (9:6) was obtained by dividing seeds into three fractions based on seed thickness. These findings indicate that similarly to common beech seeds^59^, seeds of selected spruce species^60^ and fir seeds^37^, and hemp seeds^62^, spindle seeds should be sorted with the use of mesh screens with slotted apertures.

**Table 4:** Coefficient of variation of seed mass in three seed fractions.

Spindle species	Seed fraction	Percentage (%)	Coefficient of variation (%) of seed mass	
			Fraction	Total
Broadleaf	I (*T* ≤ 2.80 mm)	26.7	19.9	17.8
	II (*T* = 2.81–3.10 mm)	37.9	12.3	
	III (*T* > 3.10 mm)	35.4	11.8	
	I (*W* ≤ 3.20 mm)	26.7	16.4	
	II (*W* = 3.21–3.50 mm)	33.6	14.8	
	III (*W* > 3.50 mm)	39.7	12.0	
Large-winged	I (*T* ≤ 2.60 mm)	25.2	21.5	23.3
	II (*T* = 2.61–3.00 mm)	45.4	16.7	
	III (*T* > 3.00 mm)	29.4	14.5	
	I (*W* ≤ 3.00 mm)	32.8	19.8	
	II (*W* = 3.01–3.40 mm)	36.1	14.5	
	III (*W* > 3.40 mm)	31.1	14.9	
European	I (*T* ≤ 3.00 mm)	26.3	17.9	24.8
	II (*T* = 3.01–3.50 mm)	49.1	18.8	
	III (*T* > 3.50 mm)	24.6	13.9	
	I (*W* ≤ 3.50 mm)	27.2	18.0	
	II (*W* = 3.51–3.90 mm)	42.1	16.6	
	III (*W* > 3.90 mm)	30.7	17.8	
Hamilton’s	I (*T* ≤ 2.50 mm)	29.3	23.2	24.7
	II (*T* = 2.51–2.70 mm)	37.1	17.4	
	III (*T* > 2.70 mm)	33.6	13.6	
	I (*W* ≤ 2.80 mm)	31.0	23.3	
	II (*W* = 2.81–3.10 mm)	38.0	21.8	
	III (*W* > 3.10 mm)	31.0	17.1	
Winged	I (*T* ≤ 2.40 mm)	29.7	30.1	30.2
	II (*T* = 2.41–2.80 mm)	43.2	21.1	
	III (*T* > 2.80 mm)	27.1	18.9	
	I (*W* ≤ 2.70 mm)	33.1	25.5	
	II (*W* = 2.71–3.00 mm)	37.3	21.0	
	III (*W* > 3.00 mm)	29.7	23.1	

*T* thickness, *W* width.

**Figure 2:**
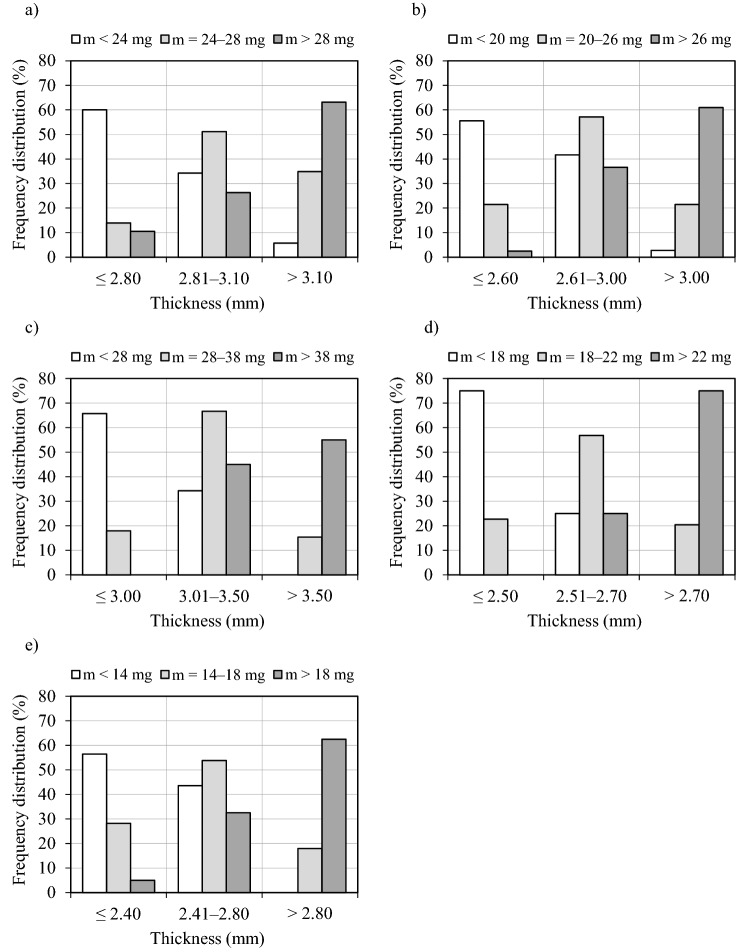
Distribution of seed thickness in spindle species: a) broadleaf, b) large-winged, c) European, d) Hamilton’s, e) winged.

The distribution of seed fractions for sorting operations based on seed thickness is presented in a histogram in Fig. 2. The thinnest seeds should be separated with a mesh screen with slotted apertures. Aperture size should be adapted individually to the processed spindle species: ≠ 2.4 mm for winged spindle seeds, ≠ 2.5 mm for Hamilton’s spindle seeds, ≠ 2.6 mm for large-winged spindle seeds, ≠ 2.8 mm for broadleaf spindle seeds, and ≠ 3.0 mm for European spindle seeds. The thickest seeds should be sorted with the use of mesh screens with slotted apertures measuring ≠ 2.7 mm for Hamilton’s spindle seeds, ≠ 2.8 mm for winged spindle seeds, ≠ 3.0 mm for large-winged spindle seeds, ≠ 3.1 mm for broadleaf spindle seeds, and ≠ 3.5 mm for European spindle seeds. A combination of two mesh screens should be applied in a sieve separator to sort spindle seeds into the following fractions:

- fraction I (thinnest seeds) containing approximately 25% to 30% of all seeds of a given spindle species—from around 56% (large-winged and winged spindle) to around 75% (Hamilton’s spindle) class 1 seeds; from around 14% (broadleaf spindle) to around 28% (winged spindle) class 2 seeds; and from 0% (European and Hamilton’s spindle) to around 11% (broadleaf spindle) of class 3 species;
- fraction II (medium-thick seeds) containing approximately 37% to 49% of all seeds of a given spindle species—from around 25% (Hamilton’s spindle) to around 44% (winged spindle) of small seeds; from around 51% (broadleaf spindle) to around 67% (European spindle) of medium-sized seeds; and from around 25% (Hamilton’s spindle) to around 45% (European spindle) of large seeds;
- fraction III (thick seeds) containing approximately 25% to 35% of all seeds of a given spindle species—from 0% (European, Hamilton’s and winged spindle) to around 6% (broadleaf spindle) of class 1 seeds; from around 15% (European spindle) to around 35% (broadleaf spindle) of class 2 seeds; and from around 55% (European spindle) to around 75% (Hamilton’s spindle) of class 3 seeds.

The sorting process is somewhat less effective when seeds are divided into three fractions based on width. However, the results of the relevant analysis were not presented because each of the three fractions would always contain seeds of all mass classes.

## Materials and methods

### Sample preparation

**Figure 3:**
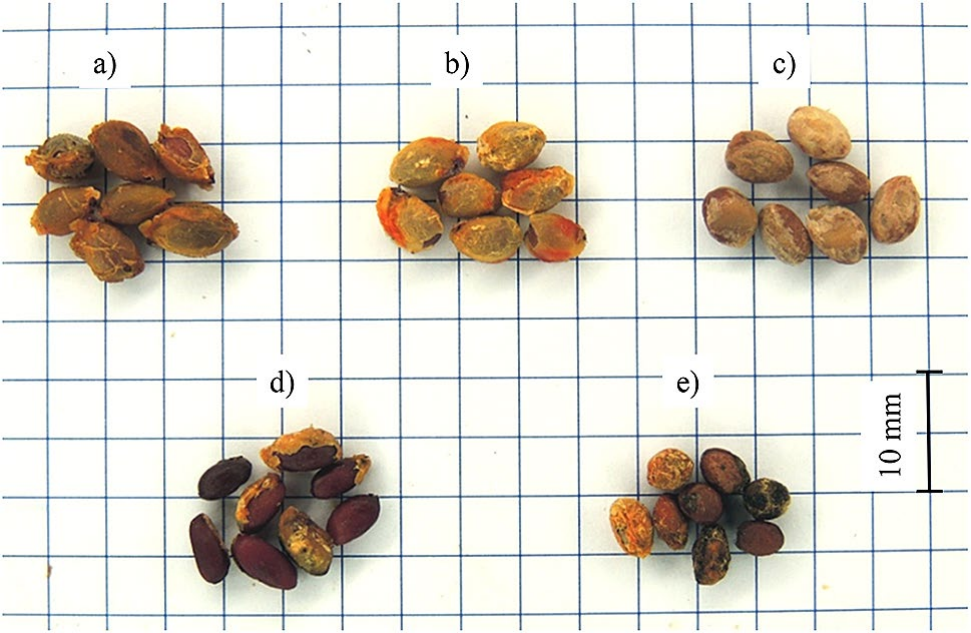
Seeds of: (**a**) broadleaf spindle, (**b**) large-winged spindle, (**c**) European spindle, (**d**) Hamilton’s spindle, (**e**) winged spindle.

The seeds of five spindle species were analyzed in this study (Fig. 3): broadleaf spindle (*Euonymus latifolius* (L.) Mill.), large-winged spindle (*Euonymus macropterus* Rupr.), European spindle (*Euonymus europaeus* L.), Hamilton's spindle (*Euonymus hamiltoniana* Wall.) and winged spindle (*Euonymus alatus* (Thunb.) Siebold.). The seeds were obtained from Dendrona (Pęcice, Poland) which distributes the seeds of forest trees and shrubs. This company is a member of the Polska Izba Nasienna organization, and it obtains seed material from reputable foreign companies and regular domestic suppliers. Each sample contained 200 g of seeds with a declared germination capacity of 60% (large-winged spindle) to 83% (European spindle) and relative moisture content of 10.2% (European spindle) to 10.8% (large-winged spindle). The acquired seeds were characterized by high purity (95–99%), but impurities were manually separated from seed lots before physical measurements. Samples containing at least 100 seeds of each spindle species were used in the analysis. Seeds were selected from lots by halving^63^. Each seed lot was divided into two portions, one portion was randomly selected and divided in half. The halving procedure was repeated to produce samples with the required number of seeds. The obtained samples contained 107 to 119 seeds. In some cases, arils were not fully detached from seeds, and they were removed manually to ensure that all measurements were performed on clean seeds.

### Physical properties

**Figure 4:**
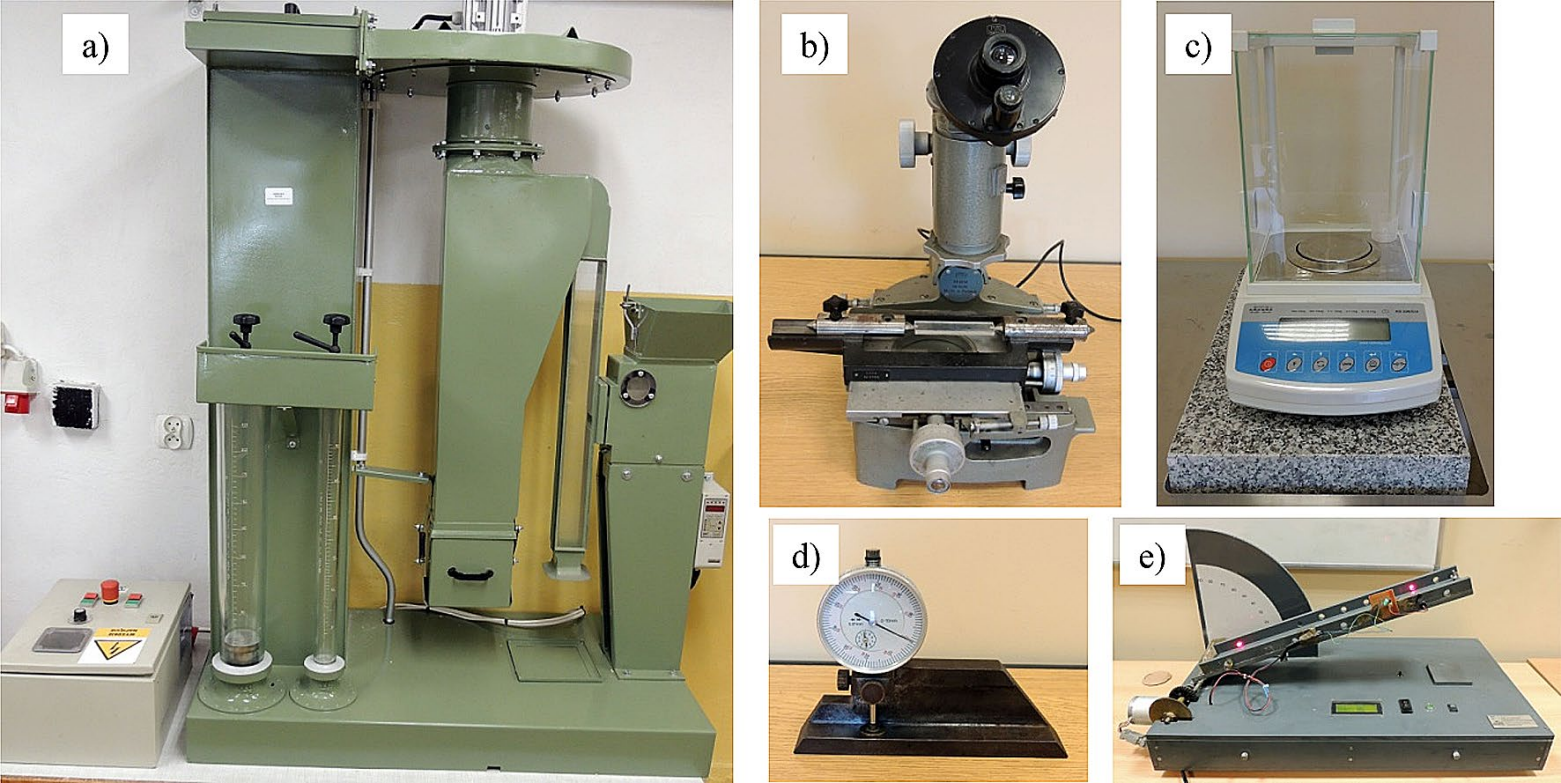
Laboratory installations: (**a**) Petkus K-293 air separator, (**b**) MWM 2325 workshop microscope, (**c**) WAA 100/C/2 laboratory scale, (**d**) dial thickness gauge, (**e**) friction plate.

Each seed was measured to determine its basic physical properties: terminal velocity (*v*), length (*L*), width (*W*), thickness (*T*), mass (*m*) and angle of external friction (*γ*). The measurements were performed with the use of the Petkus K-293 air separator (Petkus Technologie GmbH, Wutha-Farnroda, Germany), MWM 2325 workshop microscope (PZO, Warsaw, Poland), dial thickness gauge designed by the authors, WAA 100/C/2 laboratory scale (Radwag, Radom, Poland), and an inclined friction plate with surface roughness *Ra* = 0.48 µm (Fig. 4). The measurements were conducted with an accuracy of 0.11 m s^−1^, 0.01 mm, 0.01 mm, 0.1 mg and 0.1°, respectively. The measuring procedure was described previously by Kaliniewicz et al.^64^. The angle of external friction was expressed by the average result of two measurements where the seed’s longitudinal axis was positioned parallel and perpendicular to the direction of movement on the inclined plate.

The measured values were used to calculate *T/W*, *T/L* and *W/L* aspect ratios^35^, geometric mean diameter *D*, sphericity index *Φ*^65^:

1 $$D={\left(T\cdot W\cdot L\right)}^{\frac{1}{3}}$$

2 $$\Phi =\frac{D}{L}\cdot 100$$

and specific mass *m*_*D*_^60^:

3 $${m}_{D}=\frac{m}{D}$$

The seeds of each spindle species were sorted into three (nearly equal) mass classes: small (class 1—light seeds), medium (class 2—seeds with average mass) and large (class 3—heavy seeds). Seeds were divided into classes based on their mass which was measured to the nearest 1 mg. In the separation process based on seed thickness and width, seeds were divided into three nearly equal fractions based on measurements that were conducted to the nearest 0.1 mm.

### Statistical analysis

The results of seed measurements were processed statistically in Statistica PL v. 13.3 (StatSoft Poland Ltd., Cracow, Poland) at a significance level of α = 0.05. The normality of data distribution was checked, and descriptive statistics were used to determine the mean value and standard deviation of the measured properties. The variations in seed properties were determined by one-way analysis of variance (ANOVA). Homogeneous groups were identified by Duncan’s test. The strength of the correlations between the physical properties of spindle seeds was determined by calculating linear correlation coefficients, and the functions describing these relationships were determined in a regression analysis. The functions available in Statistica were tested, and the simplest function with a relatively high coefficient of determination (minimum 0.5) was selected.

## Conclusions

Significant similarities in all physical properties of seeds were not found between any two of the five compared spindle species. The only trait that was similar in all analyzed species was the *T/W* aspect ratio which ranged from around 88% to around 90% on average. The seeds of all spindle species differed significantly in width, mass, *T/L* aspect ratio, geometric mean diameter, sphericity index and specific mass. The highest number of similarities in the physical properties of seeds was noted in broadleaf, large-winged and Hamilton’s spindle seeds, and the smallest number of similarities—in European spindle seeds. The evaluated spindle species were arranged in the following ascending order based on the geometric mean diameter of seeds: winged spindle, Hamilton’s spindle, large-winged spindle, broadleaf spindle and European spindle.

The mass of spindle seeds was significantly correlated with basic seed dimensions. Seed mass was less correlated with terminal velocity, and it was least correlated with the angle of external friction. The relationships between seed mass vs. seed thickness and seed width were characterized by high values of the coefficient of determination which reached 0.72 and 0.73, respectively. The results were well described by linear equations. The identified correlations between seed attributes can be helpful in planning and performing seed processing operations and configuring processing machines to conform with the values of a given physical parameter when only a trait that is strongly correlated with this parameter can be measured.

Spindle seeds should be sorted with the use of mesh screens with slotted apertures. Aperture size should be adapted individually to the processed species, and it should range from ≠ 2.7 to ≠ 3.5 mm in the top screen, and from ≠ 2.4 to ≠ 3.0 mm in the bottom screen. Seeds will be separated into three size fractions characterized by more uniform distribution of seed mass than non-processed seeds. Seeds for sowing in forest nurseries should be sorted to obtain seedlings with a more uniform growth habit.
